# Optimization of PTFE Coating on PDMS Surfaces for Inhibition of Hydrophobic Molecule Absorption for Increased Optical Detection Sensitivity

**DOI:** 10.3390/s21051754

**Published:** 2021-03-04

**Authors:** Junyi Yao, Yiyang Guan, Yunhwan Park, Yoon E Choi, Hyun Soo Kim, Jaewon Park

**Affiliations:** 1 School of Microelectronics, Southern University of Science and Technology, Shenzhen 518055, China; yaojy@mail.sustech.edu.cn (J.Y.); 11510143@mail.sustech.edu.cn (Y.G.); 2 Division of Environmental Science & Ecological Engineering, Korea University, Seoul 02841, Korea; sug4393@korea.ac.kr (Y.P.); yechoi@korea.ac.kr (Y.E.C.); 3 Daegu Research Center for Medical Devices and Rehabilitation, Korea Institute of Machinery and Materials, Daegu 42994, Korea; 4 Department of Electronic Engineering, Kwangwoon University, Seoul 01897, Korea

**Keywords:** PDMS surface modification, PTFE coating, fluorescence absorption inhibition, Nile red

## Abstract

Polydimethylsiloxane (PDMS) is a polymer widely used for fabrication and prototyping of microfluidic chips. The porous matrix structure of PDMS allows small hydrophobic molecules including some fluorescent dyes to be readily absorbed to PDMS and results in high fluorescent background signals, thereby significantly decreasing the optical detection sensitivity. This makes it challenging to accurately detect the fluorescent signals from samples using PDMS devices. Here, we have utilized polytetrafluoroethylene (PTFE) to inhibit absorption of hydrophobic small molecules on PDMS. Nile red was used to analyze the effectiveness of the inhibition and the absorbed fluorescence intensities for 3% and 6% PTFE coating (7.7 ± 1.0 and 6.6 ± 0.2) was twofold lower compared to 1% and 2% PTFE coating results (17.2 ± 0.5 and 15.4 ± 0.5). When compared to the control (55.3 ± 1.6), it was sevenfold lower in background fluorescent intensity. Furthermore, we validated the optimized PTFE coating condition using a PDMS bioreactor capable of locally stimulating cells during culture to quantitatively analyze the lipid production using *Chlamydomonas reinhardtii* CC-125. Three percent PTFE coating was selected as the optimal concentration as there was no significant difference between 3% and 6% PTFE coating. Intracellular lipid contents of the cells were successfully stained with Nile Red inside the bioreactor and 3% PTFE coating successfully minimized the background fluorescence noise, allowing strong optical lipid signal to be detected within the PDMS bioreactor comparable to that of off-chip, less than 1% difference.

## 1. Introduction

One of the most popular polymer materials used in microfluidic systems is polydimethylsiloxane (PDMS) due to its numerous advantages including convenience of fabrication, biocompatibility, optical transparency, and gas permeability [1,2,3,4]. The intriguing porous matrix structure of PDMS endows the high gas permeability, which is ideal for cell culture studies using microfluidic devices [5,6,7,8,9]. Unfortunately, the porous matrix nature of PDMS can be a bottle neck in some applications such as the cell/proteomic analysis, or drug discovery applications that utilizes small hydrophobic molecules as PDMS can absorb such molecules [10,11,12,13,14]. Commonly used hydrophobic fluorescent molecules (e.g., Nile red, quinine, BODIPY, and Rhodamine B) are also absorbed by PDMS when flowing inside microchannels for staining target analyte, resulting in very strong background fluorescence noise that significantly lowers the signal to noise ratio (SNR) and makes it difficult to optically detect or analyze samples [15,16,17,18,19,20]. In order to overcome such limitations, various approaches have been proposed to prevent the absorption of such molecules by modifying the PDMS surface but had limitations such as swelling/dissolving of the devices, low thermal stability, or short-time prescriptions [21,22,23]. Roman et al. [21] employed the transition metal sol–gel method that entailed the metal alkoxides to diffuse into the PDMS channel wall and hydrolyzed with water vapor to form metal oxide surfaces for preventing the absorption of the small hydrophobic molecules. Although useful, the precursor chemical solution used in the sol–gel method caused swelling/dissolving of PDMS that resulted in modified PDMS surfaces to crack. Another approach suggested by Sasaki et al. [22] was to coat the PDMS surfaces with a paraffin wax layer to resist the small molecule absorption; however, thermal stability of the paraffin wax was poor and the biocompatibility of this approach was not clearly verified. Kim et al. [23], used Bovine Serum Albumin (BSA), having no biocompatibility issues, to coat the surface of PDMS microchannels to minimize the absorption of Nile red. BSA was effective in reducing the Nile red absorption but the dye could still be absorbed and the effect of BSA rapidly decreased over time.

Polytetrafluoroethylene (PTFE), popularly known as the brand name Teflon^®^, is a material broadly used in many industrial sectors and is also called “King of Plastics” [24,25,26,27,28]. PTFE is well known for its good chemical, thermal, electrical stability, and low friction that draw interests of many researchers [29,30]. In recent years, applications of PTFE are extended over many different areas such as chemical and medical industries, automotive, food, and others [31,32,33]. Despite such broad applicability, only a few researches that utilize PTFE coating to modify PDMS surfaces were reported and applications as well as characterization were rather limited [34,35,36,37,38].

In our study, we have coated the PDMS microchannel surfaces with PTFE to inhibit the absorption of hydrophobic fluorescent molecules for minimizing background fluorescence noise to increase the signal to noise ratio (SNR) of the target analyte for optical detection. Four different concentrations of PTFE were coated to PDMS microchannel surfaces to find the optimized concentration that allows for sensitive fluorescent signal detection comparable to conventional off-chip analysis as too high concentration coating can result in reduced gas permeability along with reduced optical transparency. The optimization of the PTFE coating was characterized using a fluorescent dye, Nile red (9-diethylamino-5H-benzo[a]phenoxazine-5-one) that shows high absorption to PDMS [39]. The optimized PTFE coating concentration was validated by performing an on-chip Nile red staining with *Chlamydomonas reinhardtii* (*C. reinhardtii*) CC-125 cells inside a newly developed PDMS bioreactor and analyzing the intracellular lipid content fluorescence intensity profile. We believe our optimized PTFE coating results, comparable to off-chip conventional assay methods, can be a valuable reference for other hydrophobic fluorescent staining-based microfluidic devices as it can significantly increase the SNR of the target analyte for optical detection and can be performed simply for even devices having microfluidic pneumatic valve structures.

## 2. Materials and Methods

### 2.1. PDMS Bioreactor Design

**Figure 1:**
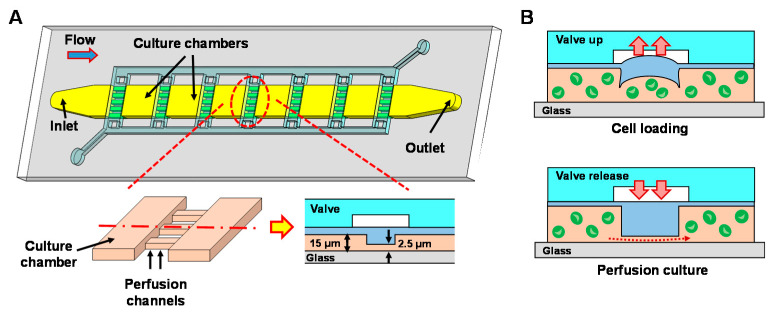
(**A**) A schematic illustration of the polydimethylsiloxane (PDMS) bioreactor. Insets: Close-up and cross-sectional view showing two culture chambers connected via shallow perfusion microchannels. (**B**) Operating principle of the valves showing cell loading process (top) and culture medium perfusion process (bottom).

The PDMS microfluidic bioreactor used for PTFE coating is designed as a microalgae culture platform capable of analyzing their growth and lipid production (Figure 1). The bioreactor is composed of 10 culture chambers (width: 400 μm, length: 400 μm, height: 15 μm) and arrays of perfusion channels (width: 25 μm, length: 400 μm, height: 2.5 μm). Neighboring culture chambers are connected via 16 perfusion channels, where pneumatically actuated valves are integrated on top of the perfusion channels (Figure 1A—insets). Perfusion channels are designed to be shallower than the target cell size and this allows all culture chambers to be fluidically connected even when the valves are closed, while confining cells inside each culture chamber. As shown in Figure 1B, perfusion channels are lifted up by the valves during the cell loading to allow cells to pass through the region and to be loaded into each chamber. After cell loading, valves are released and cells are confined inside each chamber. The perfusion channels not only allow nutrient supply to all chambers during culture but also confine cells within the chambers during biomolecular treatments such as staining of cellular lipid contents with fluorescent dyes (e.g., Nile red). The unique configuration of the bioreactor can provide screening capability where microalgal cells in each chamber can be tested under different stimulation conditions (e.g., light intensity, wavelength) in parallel.

### 2.2. Fabrication

**Figure 2:**
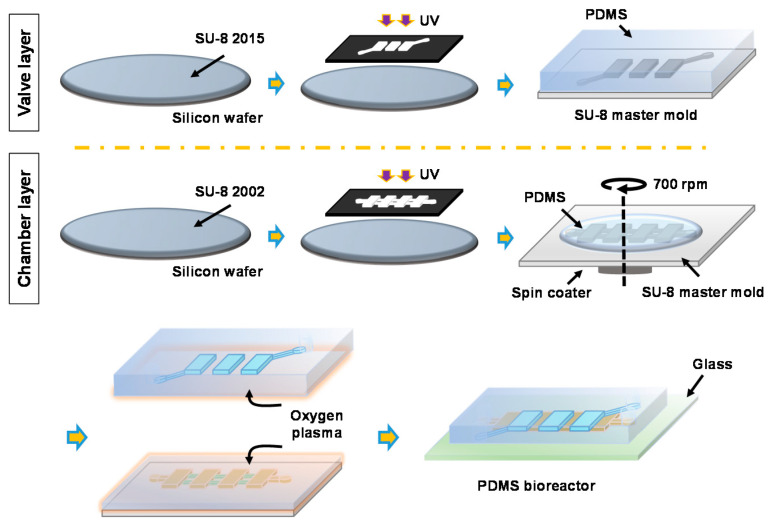
Fabrication process of the PDMS bioreactor.

The PDMS device was fabricated by the conventional soft lithography technique. Master molds for both the valve layer (height: 80 μm) and the cell culture layer were prepared by the photolithography process with SU-8^TM^ (Microchem Corp., Westborough, MA, USA) negative photoresists. For the cell culture layer, two-step photolithography was performed to obtain structures having two different heights (culture chamber height: 15 μm, perfusion channel height: 2.5 μm). Overall fabrication process is summarized in Figure 2. To facilitate the PDMS replication, both master molds were treated with trichloro(*1H,1H,2H,2H*-tridecafluoro-n-octyl)-silane (≥97% [GC], Tokyo Chemical Industry Co., Tokyo, Japan) for 10 min. The PDMS valve layer was replicated from the master mold by casting 25 g of PDMS (Sylgard 184, Dow Corning, Midland, MI, USA) mixed with curing agent at 10:1 ratio. For the chamber layer, 15 g of PDMS (mixing ratio with curing agent = 10:1) was poured onto the master mold, degassed to remove bubbles, then spin-coated at 700 rpm for 60 s to obtain a PDMS membrane with the thickness of 100 μm. Both the chamber layer and the valve layer were cured for 40 min inside an 80 °C oven. Holes for the valve layer were then punched with a 0.5 mm hole-puncher (Harris Uni-Core, Ted Pella, Inc., Redding, CA, USA). After the oxygen plasma treatment for 60 s (PDC 002, Harrick Plasma, Ithaca, NY, USA), the valve layer was aligned and bonded with the chamber layer under a stereomicroscope (NSZ608T, Jiangnan, China). Finally, the assembled PDMS device was bonded to a glass slide (76.2 × 25.4 mm^2^, Sail Brand Co., Zhejiang, China) using the oxygen plasma treatment. During the bonding process, negative pressure was applied to the valves (lifted-up) to prevent the perfusion channel area from permanently bonding to the glass slide. The PDMS bioreactor was immersed in deionized (DI) water to prevent bubble entrapment in the subsequent operations.

### 2.3. PTFE Coating on PDMS Bioreactor

**Figure 3:**
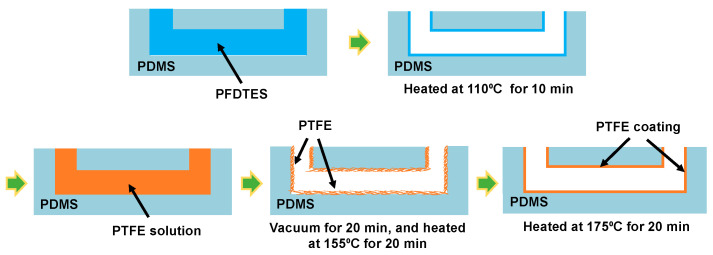
Polytetrafluoroethylene (PTFE) coating process for PDMS microchannels.

Similar protocol was used for coating as previously reported (Figure 3) [34]. First, *1H,1H,2H,2H*-perfluorodecyltriethoxysilane (PFDTES, 97%, Sigma Aldrich Inc., St. Louis, MO, USA) solution was added to the microchannels in the PDMS bioreactor for 30 min and the device was heated at 110 °C for 10 min to coat the microchannel surfaces with PFDTES layer. Due to the presence of the fluorinated carbons, PFDTES is a fluoroalkyl-silane (FAS) with low surface energy and high wettability, which helps to promote the adhesion between the PDMS surface and PTFE. We expect this helps the PTFE coating to remain intact even during the valve operation. After the PFDTES coating, PTFE solution (AF 1600, DuPont^TM^ Corp., Wilmington, DE, USA) diluted at the concentration of 1%, 2%, 3%, and 6% (*v*/*v*) in FC-40 (Sigma Aldrich, St. Louis, MO, USA) was introduced into the microchannel. PTFE solution filled device was placed inside a vacuum chamber (P = −20 kPa) for 20 min to promote contact of PTFE to PDMS surfaces. Then, the device was incubated at 155 °C for 20 min to evaporate the fluoro-inert solvent and form a firm PTFE layer on the surface of the microchannel, followed by further heating at 175 °C for 20 min for hard baking. 

### 2.4. Cell Preparation

*C. reinhardtii*, strain CC-125, was cultured in Trisacetate-phosphate (TAP) medium (pH = 7.0) using a shaking incubator (Speed: 120 rpm) at 23 °C under 50 μmol m^−2^ s^−1^ of white fluorescence illumination with a 12-h light-dark cycle. An optical Quantum Flux (MQ 200, Apogee Instruments, Logan, UT, USA) was used to measure the light intensity. Lipid accumulation of *C. reinhardtii* was induced by centrifuging the cells at 4000 rpm for 10 min and culturing them in the TAP medium without nitrogen source (NH_4_Cl) for one week [40,41]. *C. reinhardtii* are known to induce lipid production and accumulate it inside when they are exposed to unfavorable culture environments, such as nutrient depletion, high temperature, and excessive light intensity [42,43,44]. Nitrogen-starved (N-starved) cells were harvested and prepared for on-chip (i.e., inside the PDMS bioreactor) and off-chip (i.e., on a petri dish) Nile red staining.

### 2.5. Analysis of Nile Red Absorption

For the characterization of the PTFE coating concentration, a device having straight PDMS microchannels (width: 300 μm, height: 15 μm) was used to minimize any intervention from the microchannel designs. PTFE was coated at four different concentrations (1%, 2%, 3%, and 6%) to investigate their inhibition effects on fluorescent hydrophobic molecule absorption. First, 1 µg/mL of Nile red (3.14 mM, Sigma Aldrich Inc., St. Louis, MO, USA), diluted in dimethyl sulfoxide (DMSO), was added to the microchannels. The device was then incubated for 20 min under dark light condition, followed by rinsing it with DI water three times. 

For analyzing the degree of Nile red absorption, fluorescent images of the PDMS microchannels were taken with a fluorescence microscope (Observer Z1, Carl Zeiss, Germany) equipped with a digital camera (ORCA-Flash 4.0 C13440, Hamamatsu, Japan). Line fluorescence intensity profiles of the images were measured, perpendicular to the microchannels, using NIH ImageJ software (Bethesda, MD, USA). The region of interest (ROI) was set as ±135 μm from the center of the microchannel to eliminate the peak noise signal at the PDMS channel sidewalls for more accurate analysis. A minimum of three measurements was performed using different devices to obtain the fluorescence intensity profiles. The effect of the PTFE coating with different concentrations were analyzed and compared for finding the optimal coating condition. The results were also statistically analyzed by the t-test, with *p* < 0.05 considered to be statistically significant.

### 2.6. Off-Chip Nile Red Staining of C. reinhardtii CC-125

The N-starved *C. reinhardtii* CC-125 were stained off-chip by centrifuging 1 mL of cell solution and removing the supernatant. Subsequently, 100 µL of culture medium and 5 µL of Nile red (200 µg/mL diluted in DMSO) were added to cells for the final Nile red concentration of 10 μg/mL (31.4 mM). Cells were stained for 20 min in the dark at room temperature (23–25 °C).

### 2.7. On-Chip Nile Red Staining of C. reinhardtii CC-125

Staining the lipid content of *C. reinhardtii* CC-125 with Nile red inside the PDMS bioreactor was carried out by culturing N-starved cells (4 × 10^6^ cells/mL) in the device followed by adding Nile red (10 μg/mL, 31.4 mM) and incubating for 20 min under dark light condition. Images of cellular lipid contents and chlorophyll were obtained using a fluorescence microscope. All experiments were conducted at room temperature (23–25 °C). Quantitative analysis of the stained lipid contents was performed by measuring the average fluorescent intensity of per cell area from the fluorescent images using NIH ImageJ software. 

## 3. Results

### 3.1. Optimization of the PTFE Coating Concentration

**Figure 4:**
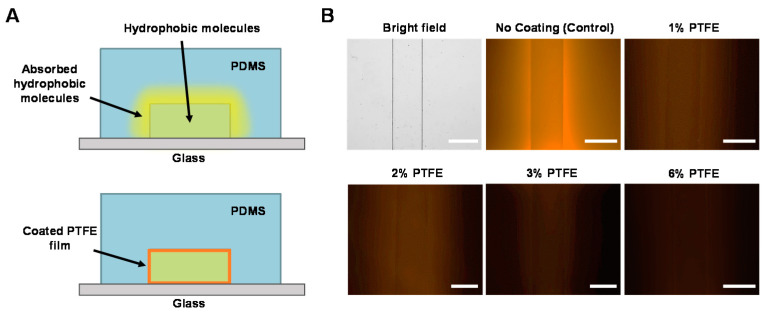
(**A**) Inhibition of hydrophobic molecule absorption on PDMS microchannel surfaces by PTFE coating. (**B**) Fluorescence images showing different levels of Nile red absorption of PDMS microchannels by different PTFE coating concentrations. Scale bars = 300 μm.

Figure 4A shows the schematic of the PTFE coating inhibiting the absorption of fluorescent hydrophobic molecules on PDMS surfaces. Fluorescent microscopic images of PDMS microchannels coated at four different concentrations of PTFE are shown in Figure 4B. It can be clearly seen that the PDMS microchannel without a PTFE coating (control) exhibiting significant absorption of Nile red, resulting in very strong background fluorescence signal even after rinse. When microchannels were coated with PTFE, absorption of the dye was reduced even at the lowest coating concentration condition (1% PTFE). 

The decrease in absorbed fluorescence intensity was obviously noticeable up to 3% PTFE coating, while difference between 3% and 6% coating was not visually identifiable. The markedly reduced absorption of the Nile red dye indicates that the PTFE coating is effective in inhibiting hydrophobic molecule absorption on PDMS, where the effectiveness is dependent on the coating concentration of the PTFE.

**Figure 5:**
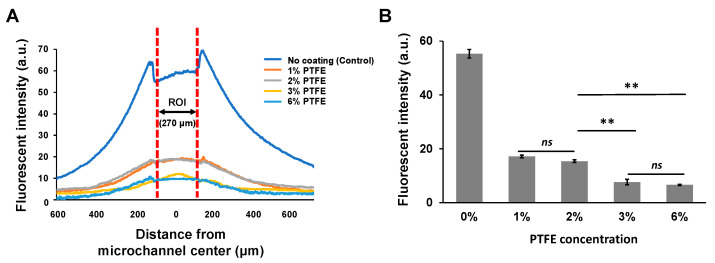
(**A**) Fluorescent intensity profile of the microchannels coated with different PTFE concentrations after 20 min of incubation with Nile red. (**B**) Average fluorescent intensity of the region of interest (ROI) with different PTFE coating concentrations (** *p* < 0.01, ns: not significant).

To further investigate the effect of the PTFE coating, Nile red absorption at different coating concentrations were quantitatively analyzed by measuring intensity profile from the fluorescence images (Figure 5A). Without the PTFE coating (control), the average absorbed fluorescence intensity within the microchannel (i.e., ROI) was 55.3 ± 1.6, which decreased down to 17.2 ± 0.5 even with 1% PTFE coating. This is more than 3.2-fold lower. Higher concentrations of PTFE coating were even more effective, where the absorbed fluorescence intensities of the 3% and 6% PTFE coatings were 7.7 ± 1.0 and 6.6 ± 0.2, respectively, both more than twofold lower than the 1% PTFE coating (Figure 5B). Comparing the absorbed fluorescence intensities of 3% and 6% PTFE coating to the control (no coating), the average intensity decreased more than sevenfold in both cases. One interesting thing we found is that although the inhibition of Nile red absorption showed an increasing tendency with higher PTFE coating concentrations, there was no significant difference between 1% and 2% PTFE coating (*t*-test, *p* = 0.6860) and between 3% and 6% PTFE coating (*t*-test, *p* = 0.6901). Nevertheless, 3% and 6% PTFE coating results showed almost two times decreased absorption intensity compared to 1% and 2% PTFE coating results. We assume that the higher PTFE coating yields better inhibition of hydrophobic molecules on PDMS but maybe in a wider concentration-step range, indicating that PTFE coating concentrations higher than 3% would have minimal additive effect. Based on our experimental results, we believe that 3% PTFE coating to be the optimal concentration for inhibiting hydrophobic molecule absorption on PDMS.

### 3.2. On-Chip Staining of C. reinhardtii Inside the PDMS Bioreactor

**Figure 6:**
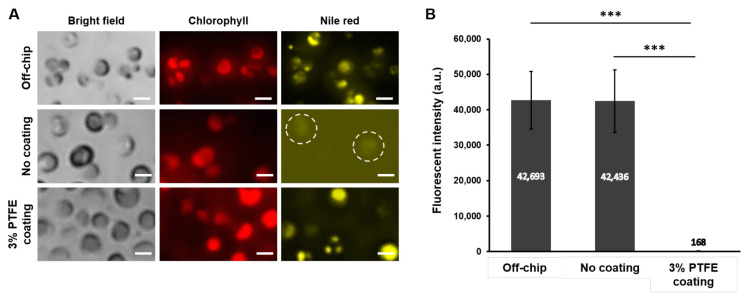
(**A**) Microscope images showing *C. reinhardtii* CC-125 stained with Nile red (yellow) and Chlorophyll autofluorescence (red) for off-chip, PDMS device without coating, and the PDMS device with 3% PTFE coating. Scale bars = 5 μm. (**B**) Average fluorescent intensity of the stained cells (*n* = 10, *** *p* < 0.001).

Finally, we assessed whether the optimized PTFE coating condition could successfully inhibit the absorption of the hydrophobic molecules to the PDMS while properly staining the biological samples inside the device for on-chip detection and analysis. As a demonstration example, *C. reinhardtii* CC-125 were loaded into the PDMS bioreactor and intracellular lipid contents were stained with Nile red. Having valves on top of the perfusion channels successfully allowed *C. reinhardtii* CC-125 to be loaded inside all culture chambers and they were confined within each culture chamber after releasing the valve. The bioreactor design, having perfusion channels, confined cells within the chamber while flowing Nile red for staining. After staining *C. reinhardtii* cells with Nile red for 20 min inside the bioreactor without the PTFE coating and with 3% PTFE coating, fluorescent intensity of the stained intracellular lipid contents was compared to conventional off-chip stained cells. Figure 6A shows the fluorescent signals of chlorophyll autofluorescence and Nile red stained intracellular lipid contents for all experimental groups (off-chip, no coating, and 3% PTFE coating). When *C. reinhardtii* were stained off-chip, their lipid signals were clearly distinguishable, and we could confirm the proper accumulation of lipid contents of the tested cells. Likewise, cells stained inside the 3% PTFE coated PDMS bioreactor also showed strong lipid content signals with almost no noticeable background fluorescence. In contrast, when cells were stained inside the PDMS bioreactor without the PTFE coating, lipid contents could be barely identified due to high background fluorescence signals resulting from PDMS absorption of the Nile red. The lipid signal intensities among different conditions were further analyzed by quantitatively comparing the average lipid fluorescent intensity (Figure 6B). The average lipid fluorescent intensity for off-chip stained cells and the cells stained inside the 3% PTFE coated PDMS bioreactor was almost the same, less than 1% difference; however, for the cells stained inside the PDMS bioreactor without PTFE coating showed significantly lower signal intensity, which was only about 0.4% compared to the off-chip and the 3% PTFE coated results. This demonstration strongly supports that the optimized coating concentration reported in this paper allows to detect and analyze optical fluorescent signals comparable to off-chip by inhibiting the absorption of hydrophobic molecules such as Nile red on PDMS devices. Figure 4A shows the schematic of the PTFE coating inhibiting the absorption of fluorescent hydrophobic.

## 4. Conclusions

The absorption of fluorescent hydrophobic molecules on PDMS microchannels significantly reduced the sensitivity of fluorescent signal detection for PDMS microfluidic devices. The absorbed fluorescence intensities of 3% and 6% PTFE coating were found to be approximately sevenfold lower than the PDMS devices without PTFE coating. They were also twofold lower than the 1% and 2% PTFE coating. Higher PTFE concentration resulted in improved inhibition effect but 3% PTFE coating was found to be the optimal concentration that minimizes the absorption as it did not show noticeable and statistical difference compared to the higher concentration. Effectiveness of the optimized coating condition (3% PTFE coating) was demonstrated by staining lipid contents of *C. reinhardtii* CC-125 with Nile red inside the PDMS bioreactor and the analysis indicated that the measured optical signals were comparable to off-chip stained results. The measured fluorescent intensity difference was less than 1%. We believe our results can provide a useful reference for developing PDMS microfluidic devices that utilize various small hydrophobic molecules.
